# Taking a Deep Breath: an Examination of Current Controversies in Surgical Procedures in Lung Transplantation

**DOI:** 10.1007/s40472-022-00367-0

**Published:** 2022-05-16

**Authors:** Gabriel Hirdman, Anna Niroomand, Franziska Olm, Sandra Lindstedt

**Affiliations:** 1 Department of Cardiothoracic Surgery and Transplantation, Skåne University Hospital, Lund University, Lund, Sweden; 2 Wallenberg Center for Molecular Medicine, Lund University, Lund, Sweden; 3 Department of Clinical Sciences, Lund University, Lund, Sweden; 4 Lund Stem Cell Center, Lund University, Lund, Sweden; 5 Rutgers Robert Wood Johnson Medical School, New Brunswick, NJ USA

**Keywords:** Lung transplantation, Extracorporeal membrane oxygenation (ECMO), Machine circulatory support (MCS), Bronchial anastomosis, Size mismatch, Delayed chest closure, Ex vivo lung perfusion (EVLP)

## Abstract

**Purpose of Review:**

This article reviews controversial questions within the field of lung transplantation, with a focus on data generated within the last 3 years. We aim to summarize differing opinions on a selection of topics, including bridge-to-transplantation, intraoperative machine circulatory support, bronchial anastomosis, size mismatch, delayed chest closure, and ex vivo lung perfusion.

**Recent Findings:**

With the growing rate of lung transplantations worldwide and increasing numbers of patients placed on waiting lists, the importance of determining best practices has only increased in recent years. Factors which promote successful outcomes have been identified across all the topics, with certain approaches promoted, such as ambulation in bridge-to-transplant and widespread intraoperative ECMO as machine support.

**Summary:**

While great strides have been made in the operative procedures involved in lung transplantation, there are still key questions to be answered. The consensus which can be reached will be instrumental in further improving outcomes in recipients.

## Introduction

The surgical approach to lung transplantation has benefited from numerous advances since the first successful procedure in 1963 [1]. As the field and perspectives have evolved, practices now reflect an increased understanding of the pathological processes involved and have improved graft longevity. Questions of what constitutes best practices have yet to be answered, resulting in a continued debate on pre-, peri-, and post-operative concerns. Here, we present a brief discussion of topics critical to lung transplantation today, reviewing the literature released in the past 3 years.

## Bridging to Transplantation

Due to the differences between the number of organs available and the demand for them, patients may wait on the transplant list for variable durations of time, with 54% of listed adults in 2019 having spent between 3 months and 2 years waiting for a transplant [2]. Patients may deteriorate while waiting for a lung transplant, and technical life support may be necessary until a suitable organ is available. This has led to the concept of “bridging to transplantation,” where critically ill patients are placed on extracorporeal membrane oxygenation (ECMO) prior to receiving a donor lung (Table 1). When considering the experiences centers have had with the implementation of bridging to transplantation, two central themes arise: the importance of high center volume, ECMO experience, and the role of ambulation for transplantation success. There has been a noted association between a lung transplant center’s volume and its mortality rates [12]. This correlation also appears to hold true when considering patients who are bridged to transplant, leading to the conclusion that with time and experience, the outcomes observed in bridged patients improve. In a 2018 study at a high-volume center in Vienna, across 71 adults with the intention to bridge, successful short-term and long-term outcomes concluded that a bridge to transplantation was appropriate in carefully selected patient populations [7•]. The group reported an intention-to-treat 1-year survival of 70% and a 5-year survival rate of 51%. At another high-volume center, Kukreja et al. reported a 68% successful transplant rate in 76 patients placed on ECMO with the intention to bridge to transplant and showed a 97% 1-year survival conditional upon discharge [8]. These outcomes demonstrate the feasibility of bridging-to-transplant and the potential successes that could be attained in centers with high volumes.

**Table 1:** Summary of reviewed literature on bridging to transplantation

Author	Type	Groups	Time span	Median time on ECMO	LAS	Survived to transplant	Ambulation	PGD 3 at 72H Post-LTx	Long term mortality	Difference in survival vs non-BTT	Conclusion
Tipograf et al. [3•] 2019	Retrospective	BTT: 121 patients	2009–2018	12d	92.2	70 (58%)	Total: 82 (68%) Transplant: 76% Non-transplant: 57%	N/A	1 year: 88% 3 year: 83%	No (log rank 0.16)	Ambulation the only independant predictor of mortality
Benazzo et al. [4•] 2019	Retrospective	BTT: 120	1998-2017	1998–2004: 2d 2005–2010: 6d 2010–2017: 5d	N/A	107 (89%)	Total: 33 (28%) 3d era: 24 (34.3%)	6 (5.6%) 28 (26.2% ungradeable)	(2010–2017) 90d: 74% 1 year: 70% 5 year: 60%	Yes (lower for bridged patients)	Marked learning curve with increased use of ECLS and technical developments
Langer et al. [5•] 2019	Retrospective	BTT: 34 non-BTT: 54	2012–2017	29d	BTT group: 92	N/A	N/A	N/A	1 year: 79% 3 year: 63%	No (*p* = 0.72)	Bridging time of less than 30 days is crucial to survival
Halpern et al. [6] 2019	Retrospective	BTT: 611	2005–2017	Low volume (LV): 19 Moderate volume (MV): 20 High volume (HV): 15	LV: 81 MV: 76.2 HV: 79.4	N/A	N/A	N/A	Overall: HR 1.46 LV: HR 2.32 MV: HR 1.58 HV: HR 1.15	Yes (log-rank *p* < 0.0001)	LTx centers that perform fewer than 35 annual transplants have a greater hazard of death
Hoetzeneckeret al. [7•] 2018	Retrospective	BTT:71 non-BTT: 1048	2006–2016	10	N/A	89%	26 (37%)	56.90%	From time of ECMO instertion 1y (first): 70% 3y (first): 63% 5y (first: 51% 1y (rtx): 42% 3y (rtx): 32% 5y (rtx): 32%	No, for patients bridged to first transplant (*p* = 0.197)	ECLS as a bridge to retransplantation should be used with caution
Kukreja et al. [8] 2020	Retrospective	BTT: 76	2010–2018	9.5	89.3	68%	21 (34%)	N/A	1 year: 86%	N/A	Supports the use of ECMO for rescue tranpslant. Identified bood type B and short stature as risk factors for failure to list
Vayvada et al. [9] 2021	Retrospective	BTT: 13	2016–2018	18.7 (3–55)	N/A	7 (54%)	N/A	3 (43%)	1 year: 57.1%	N/A	-
Hayes et al. [10] 2016	Retrospective	BTT: 279 non-BTT: 7949	2005–2010	N/A	87.84	N/A	N/A	N/A	ECMO (LV): Reference ECMO (MV): HR 0.678 ECMO (HV): HR 0.434	Yes, worse overall for BTT (log-rank *p* = 0.002).	The adverse influence of ECMO on survival is clear in low-volume centers but absent in the most experienced centers
Hayanga et al. [11] 2019	Retrospective	BTRT: 99 non-BTRT: 1861	2005–2017	N/A	64.8	N/A	N/A	N/A	1 year: 66.6%	Yes (*p* < 0.001). No significant difference when only considering the more recent era.	ECMO as a bridge to lungtransplant lead to a doubling of risk for 1-year mortality in the historical era. However, no difference in risk in the more recent era

*LTx* lung tansplantation, *BTT* bridge to transplant, *N/A* not applicable, *ECMO* extracorporeal membrane oxidation, *LAS* lung allocation score, *HR* hazard ratio, *ECLS* extracorpureal life support

For these high-volume centers, it may even be the case that the program can place riskier patients on ECMO while still preserving outcomes. In a study by Tipograf et al., though there were differences between the lung allocation scores in patients bridged to transplant on ECMO compared to those who were not, those bridged patients were found to have no significant differences in survival [3•]. This outcome is attributed to stringent protocols and patient selection. Bridge to transplant appears to still be a viable option within smaller centers, but the results are subject to more variability. In one smaller study of the thirteen patients who received preparatory ECMO, seven were successfully bridged to transplant, but only four survived past 1 year [9].

Several studies have highlighted the advantage high-volume centers appear to have relative to lower volume ones [3, 9, 10]. Hayes et al. specifically analyzed for this very effect, finding a discrepancy in outcomes when examining the volume of a center [10]. It was determined there was a post-transplant hazard associated with low volume centers, in contrast with the finding of no adverse influence of ECMO in high volume ones. In another analysis of center volume, Halpern et al. examined center-stratified outcomes to observe again that high volume centers with bridge-to-transplant had similar survival rates to non-bridged patients [6]. This is an effect of not just case volume, but also time. At a single high-volume center, when bridge-to-transplant patients were allocated to distinct eras, Benazzo et al. noted an improvement in short-term outcomes over time and saw that ECMO bridging had similar long-term survival to non-bridged patients [4•]. As outcomes improve with time and experience, the question then transitions to how long a patient can be on ECMO. Langer et al. found that among their bridge-to-transplant cohort, survival rates improved when ECMO was kept to a maximum of 29 days [5•]. Again, 1-year survival rates among the ECMO support group were similar (79%) to those without ECMO support (86%). Throughout these studies, the results help to validate the hypothesis that experience may be key to ensuring good outcomes among bridged patients. To that end, the authors of the study themselves note that the center gained significant ECMO experience managing adults with ARDS, which might have contributed to the success achieved in ECMO bridge-to-transplant [5•]. With this insight, the high use of ECMO during the current COVID-19 pandemic may translate into increased center experience. This could mean future improved survival for patients bridged to transplant. Indeed, mainly positive outcomes have been reported on COVID-19 patients bridged to lung transplant [13–15].

While results over time may have improved when considering bridge to a first transplant, a firm conclusion has not been reached on placing patients for bridging to retransplant. In the Vienna study, in the 16% of patients who were bridged to retransplantation out of the entire bridging group, there was a decrease in survival compared to the first time transplants group, leading to a call for caution by the authors [7•]. When Hayanga et al. examined 99 patients who were bridged, there was a greater incidence of prolonged ventilation and reduced 1-year survival in those bridged to retransplantation compared to first transplant (67% vs. 83%) [16••]. Other schools of thought have suggested that ECMO could even be a contraindication to retransplantation, with the caveat that survival rates have improved with time [17]. Within this discussion lies a debate on ethics. As eloquently explained by Hayanga, re-operative lung transplantation raises a moral quandary of how one can justify the distribution of a precious resource repeatedly to one patient and not another as both waits on the same list [16••]. This argument might not, however, hold as well for younger patients.

Moral dilemmas, such as whether to place patients on ECMO in the first place, become less relevant as we gain more information on practices which contribute to success. One factor rising in prominence in recent years has been the role of ambulatory bridging. According to Hoetzenecker et al., the use of ECMO enabling the patient to participate in physical therapy pre-transplantation may prevent deconditioning of the patient [7•]. The loss of strength that occurs in a patient spending time in the intensive care unit correlates with increased morbidity and mortality [18]. While most agree that ambulation can contribute to post-operative success, Kukreja et al. note that half of the successfully bridged patients in their cohort were non-ambulatory [8]. However, when examining factors that lead to a successful bridge-to-transplant, several studies highlighted ambulation as an independent predictor for survival [3, 4].

All the possibilities and potential impact of ECMO bridge to transplant raises the question: where should the field bridge to next? An unresolved question is which patient population we should select. How conservative should patient selection be when bridging? Tipograf et al. showed good outcomes as they stringently delisted patients who failed to perform but had a lower percentage of patients reach transplantation (56%) [3•]. Is a more conservative approach to patient selection justified when considering the use of marginal donor organs in bridge-to-transplant recipients?

## Intraoperative Machine Support

Extracorporeal life support (ECLS) should be considered in more than just the lead-up to transplantion. Discussion within the field revolves around the type and superiority of machine support (MSC) — if any at all — that should be used intraoperatively (Table 2). Patients may be placed either on cardiopulmonary bypass (CPB) or ECMO or alternatively be off-pump with one-lung ventilation (OLV) conducted sequentially. These three options come with their own advantages and disadvantages. The tradeoff for hemodynamic stability in mechanical circulatory support (MCS) is the risk of cannulation, heparinization, and the known effects of non-physiological shear stress and artificial surfaces [25, 26]. While sequential OLV avoids these pitfalls, hemodynamic instability may ensue, or there may be severe hypotension following pressure on the heart [25]. Other points of concern include the high FiO2 needed in reperfusion which has been shown to be a risk factor for PGD development [27]. The use of CPB in a study by Diamond et al. was identified as an independent risk factor for severe PGD [27]. Opinions are divided, with some who report that there can be similar post-operative outcomes between off-pump cases which required emergency conversion to CPB and elective “on-pump” [22]. Despite this, in Mohite et al.’s study of 302 transplants, patients who were off-pump were found to have better PaO2/FiO2 ratios and shorter time on ventilation, in the ICU, and hospital stay.

**Table 2:** Summary of reviewed literature on intraoperative extracorporeal machine support

Author	Type	Time span	Number of patients included	Median LAS	Median transfused packed RBCs	PGD3 at 72H	Median length of hospital stay	Survival	Conclusion
Loor et al. [19]	Retro- and prospective	2016–2020	CBP (*n* = 157) ECMO (*n* = 273) Off-pump (*n* = 422)	CPB: 47.8 ± 15.7 ECMO: 50.2 ± 19 Off-pump: 39.5 ± 11.4	N/A	CBP: 42.7% ECMO: 28.9% Off-pump: 12.1%	Off-pump: shortest postoperative length of stay	1 year survival: CBP: 84% ECMO: 84% Off-pump: 91%	Off-pump strategy should be the first choice when the surgeon believes it is safe and feasible. In cases in which ECLS use is preferable, the choice of ECMO over CPB when feasible may reduce the risk of severe PGD
Hoetzenecker et al. [20••] 2020	Prospective	2016–2018	VA-ECMO (*n* = 159)	N/A	(Mean ± SD) Mean: 4.2 ± 3.4	*n* = 2 (1.3%)	Median: 23d (18–34d IQR)	90d mortality: 96.9% 1y survival: 86%	Routine use of intraoperative ECMO resulted in excellent outcomes regarding PGD and survival
Ius et al. [21] 2020	Retrospective	2010–2019	ECMO (*n* = 311) Off-pump: (*n* = 826)	ECMO: 42.3 (Q1 35.3; Q3 63.3) Off-machine: 34.8 (Q1 32.8; Q3 38.9)	ECMO: 4 (Q1 2; Q3 7) Off-pump: 2 (Q1; Q3 3)	ECMO: *n* = 46 (14.9%) Off-pump: *n* = 12 (1.5%)	ECMO:28d (22–51) Off-pump: 22d (21–26)	ECMO (3y, 5y, 8y): 74%, 67%, and 57% Off-pump (3, 5, 8y): 83%, 73%, and 64%	ECMO allows transplantation of increasingly complex recipients. ECMO shows poorer early graft survival compared to off-machine. No differences in long-term complications were found
Mohite et al. [22] 2016	Retrospective	2007–2013	Off-pump (*n* = 86) CPB (*n* = 162) Conversion (*n* = 54)	N/A	N/A	N/A	Off-pump: 24d (19;40) CPB: 38d (25;55) Conversion: 38d (22;57)	Off-pump (1y, 3y, 5y): 94.4%, 78.1%, 51.4% CPB (1y, 3y, 5y): 86.9%, 74.3%, 65.3% Conversion (1y, 3y, 5y): 61.9%, 39.8%, N/A	Better early post-operative outcomes and early survival with an off-machine strategy for LTx rather then an elective strategy
Hashimoto et al. [23] 2018	Retrospective	2007–2016	BTT on VV ECMO and intraop: VV-ECMO (*n* = 20) VA-ECMO (*n* = 11) CPB (*n* = 3)	N/A	VV-ECMO: 5 (IQR 4–9) VA-ECMO: 8 (IQR 6–13)	N/A	N/A	VV-ECMO (30d, 90d): 100%, 100% VA-ECMO (30d, 90d): 100%, 91%	There should be a low threshold for changing to VA-ECMO in VV-ECMO bridged patients
Hoetzenecker et al. [24] 2018	Retrospective	2010–2016	w/o ECMO, Group I (*n* = 116) intraop ECMO. Group II (*n* = 343) intraop+prolonged ECMO, Group III (*n* = 123)	N/A	(Mean ± SD) Group I: 2.3 ± 1.2 Group II: 4.1 ± 2.6 Group III: 5.6 ± 4.1	Group I: 3.4 % Group II: 3.3% Group III: N/A	Group I: 21d (1–117) Group II: 22d (1–190) Group III: 33d (7–155)	Group I (1y, 3y, 5y): 82%, 76%, 74% Group II (1y, 3y, 5y): 91%, 85%, 80% Group III (1y, 3y, 5y): 84%, 81%, 76%	Intraoperative preemptive ECMO results in low PGD rates and superior survival compared to transplantation without ECMO

*ECMO* extracorporeal membrane oxygenation, *CPB* cardiopulmonary bypass, *VA-ECMO* venoarterial extracorporeal membrane oxygenation, *VV-ECMO* venovenous extracorporeal membrane oxygenation, *RBCs* red blood cells, *LAS* lung allocation score, *W/O* without, *Intraop* intraoperative, *N/A* not applicable, *IQR* interquartile range, *Q1* 1st quartile, *Q3* 3rd quartile, *d* day, *y* year, *PGD* primary graft dysfunction, *LTx* lung transplantation

When considering the application of ECMO, some advocate its use in all patients regardless of indication. Under a guideline of routine application of intraoperative ECMO to all patients within the Vienna center, remarkably low percentages of PGD were reported, with only a 10% incidence of any grade of PGD 1–3 [20••]. Two-year survival was reported at 86%, and the authors attributed the success to ECMO’s ability to control flow to reperfuse the graft gradually. Of note, there are potential limitations to such widespread application. The risk of bleeding, especially in heparinized patients, is a non-negligible threat, with 6 of the 13 patients who additionally received post-operative ECMO requiring reoperation for bleeding. Nevertheless, the takeaway from the prospective trial from the Vienna group is that ECMO prolongation should be implemented to avoid severe PGD. This follows an earlier publication by the same group, which retrospectively examined the discrepancies between ECMO use and no intraoperative mechanical support at all [24]. Thromboembolic events occurred in 9 patients who received peripheral ECMO. There were cerebral ischemic events in two patients on prolonged ECMO post-operatively. Nevertheless, the authors concluded that over their 15-year experience using ECMO as their standard MCS device, their widespread and preemptive use of ECMO had resulted in lower rates of PGD and better survival.

Considering those patients already on ECMO as a bridge to transplantation, there should be further studies on how to manage these patients intraoperatively. Hashimoto et al. found no comparable differences between venoarterial (VA) and venovenous (VV) ECMO following bridging with VV-ECMO [23]. The only apparent yet statistically insignificant difference was an increased need for transfusion following a switch to VA. The requirement of arterial cannulation with increased use of heparin in VA-ECMO carries its own set of risks, and the transfusions these patients may require are linked to a higher risk of primary graft dysfunction with increased mortality [27, 28] Other benefits outlined in Hashimoto et al.’s work are theoretical and would require greater investigation, yet the main conclusion of considering a low threshold to conversion between ECMO types is reached by finding comparable results between the two.

The superiority of ECMO is not a universally held opinion. A retrospective review of intraoperative CPB by Taka et al. reported that CPB in patients receiving lungs from extended criteria donors could exhibit favorable outcomes at 87% survival 1-year post-transplant and 81% at 5-years [29]. The ability of this group to remain comparable to transplants from standard criteria donors was attributed to protective allograft reperfusion. A strategy of elective CPB was pursued in this study to avoid the increased pulmonary artery pressure (PAP) that can occur in an implanted lung when off-pump. High PAP can lead to acute right-sided heart failure and hemodynamic collapse. Elective CPB also avoids the potential damage from high FiO2 needed during reperfusion. Arguments against ECMO included the influence a variable patient circulatory volume can have on ECMO flow and an inability to perform open pulmonary artery surgery if needed.

In a meta-analysis of over seven studies encompassing 785 patients, there were no significant differences between CPB and ECMO groups, with the caveat that there was a non-statistically significant trend of increasing mortality in those with CPB [30]. There was an increase in PGD rates among the CPB group, as well as increased bleeding. Furthermore, ECMO patients had fewer renal and pulmonary complications. However, based on ICU and hospital lengths-of-stay, the authors of the meta-analysis still came to a recommendation favoring ECMO use.

An important comparison is how MCS performs relative to off-pump transplantation. In Ius et al.’s study comparing 311 patients with ECMO to the 826 without, there were no significant differences in 5-year survival or chronic lung allograft dysfunction between the groups [21]. This excluded CPB use and corrected for patients surviving to discharge, as the authors described that ECMO patients had higher pre-transplant surgical risk profiles. Placing the patients off-pump reduces the exposure to risks that come with ECMO use, such as the risk of hypotension and desaturation due to a lack of venous reservoir and the potential for stopped flow following obstruction of the cannula. Further downsides of ECMO include the need for cell saver to retransfuse lost blood, increased IV fluid administration, and poor surgical visibility [25, 29]. Considerations might need to be made with the underlying diagnosis of the patient in mind, as studies have shown that the use of CPB in cystic fibrosis patients led to increased transfusion [31]. In another study of pediatric and adult cystic fibrosis transplants, the use of CPB was an independent predictor of mortality [31, 32]. Recently, a retro- and prospective, multicenter trial by Loor et al. from eight high-volume centers compared PGD3 rates at 48 or 72 h with CPB, ECMO, and off-pump. The early results of the trial, encompassing 852 transplants, indicate an odds ratio for developing PGD3 of 1.89 when comparing CPB vs. ECMO and as high as 4.21 when comparing CPB vs. off-pump [19].

Innovations to machine circulatory support may decrease the incidence of post-surgical complications, such as PGD. In studying a new dual-lumen single cannula in VV-ECMO, Budd et al. report on a case of pulmonary hypertension in which they hypothesize this cannula could lead to less complicated placement, decannulation, and decreased risk of infection [33]. Hybrid devices may prove to be an innovative new angle to pursue, with novel circuits being reported on in smaller studies [34, 35].

## Bronchial Anastomosis

In 2018, an ISHLT working group published a consensus statement on airway complications after lung transplantation [36]. The working group summarized known risk factors for airway complications (ACs) such as right-sided anastomoses, organ preservation techniques, and microbiologic contamination. Subsequently, a new grading system for airway complications was proposed which involved categorizing the complications based on pathophysiology. Types were divided between ischemia and necrosis, dehiscence, stenosis, and malacia and then further graded based on location and extent. One of the first large-scale studies published to use the new grading system was Schweiger et al. in 2020 [37]. Their retrospective study examined 1555 patients whom all underwent the single running technique rather than the more commonly used approach of single running on the membranous portion and single stitches on the cartilaginous portion [38]. In total, 45 patients suffered airway complications (or about 2% per anastomosis), and 82% of these could be classified according to the new ISHLT criteria, with the most common being stenosis. Although no instances were reported in their rather large cohort, the authors acknowledge the risk of purse stringing. The Vienna group concluded that a single-running technique was both time-efficient and associated with an extremely low incidence of anastomotic complications and could be used as a standard technique. However, this interpretation was challenged by Chang et al. who argued that the data were derived from a highly experienced group using the same technique for over two decades and that ultimately the choice of anastomosis should remain dependent on the surgeon’s judgment [39].

In order to minimize the portion of the airway solely supplied by the bronchopulmonary collaterals, surgeons have shortened the donor bronchus to about one or two rings above the lobar carina [40]. Recently, some authors have argued that an even shorter donor bronchus at the level of the bronchial carina could be beneficial [41].

The large disparities reported across the literature in rates of ACs continue to be a concern. A retrospective study by Patoir et al. in 2020 identified an AC prevalence of 23% in their 6-year cohort composed of 121 lung transplants [42]. Patoir et al. identified that their high rates of AC might be explained by their use of the organ preservation solution Celsior (IGL Group, Lyon, France) instead of Perfadex (XVIVO Perfusion AB, Göteborg, Sweden). They hypothesize that Celsior could potentially lead to less severe yet more frequent rates as none of those complications mandated surgical intervention. Another study by Chamogeorgakis et al. found an incidence rate of 5% of ACs among 460 bronchial anastomoses using a more traditional running-single suture technique [43]. Four patients could be managed conservatively, eight endoscopically with stents, and four surgically. Two patients had to be retransplanted due to severe distal airway stenosis.

## Size Mismatch

For the surgeon, a crucial detail to consider is whether the size of the donated graft matches the capacity of the recipient to hold it. Misalignment of the recipient and donor lung characteristics can lead to “size mismatch,” which is important given the consequences this may have on patient outcomes. There are reports that an oversized lung may lead to ventilatory dysfunction as well as atelectasis and hemodynamic instability [44]. Others have remarked that an oversized graft comes with a decreased risk for PGD grade 3 [45]. The first obstacle in evaluating a size mismatch is determining the methodology for measurement. Commonly, the ratio between the donor and recipient predicted total lung capacity (pTLC) has been utilized [44, 46]. While larger donor lungs may reduce the risk of PGD, the disease etiology must be considered. In chronic obstructive pulmonary disease (COPD) patients, the actual TLC (aTLC) is increased compared to the pTLC, which could hinder the universality of using the pTLC ratio [47]. This would suggest that the aTLC should be used whenever possible, particularly for recipients for whom the information is likely available.

To consider another form of size and potential mismatch measurement, Li et al. suggest a chest x-ray to determine patient aTLC. They observed a relationship between the ratio of the donor to recipient x-rays and PGD incidence rates [47]. The authors referred to x-ray lung height and noted that when considering lung height ratios greater than 1, there was a significantly higher percentage of those who had PGD grade 3 (24%) relative to ratios less than 1 (11%). The benefits of this metric would be its simplicity: off a set of x-rays, lung dimensions could be easily measured and could represent an accurate method for determining relative graft size. Other alternatives to the pTLC ratio have also been suggested, including calculating lung volume using CT images [48]. This approach benefits from the availability of donor CT scans and may facilitate the ease of calculating a mismatch. Jung et al. have compared the volumes derived from CT images and referenced them to values obtained through plethysmography and pulmonary function testing, finding that CT volumes either had a similar or better correlation to the total lung capacity than pTLC [48].

Surgeons who find that the donor’s lung is inappropriately oversized must then consider the outcomes following a resection. Wedge and lobar reductions could increase the pool of available donor organs, which would reduce the wait times of listed potential recipients and reduce wait-list mortality. While the graft might not be the ideal for the recipient, it would likely be preferable to no transplant at all. In a study of volume-reduction surgery, Montoya et al. analyzed downsizing larger lungs achieved by anatomic lobectomies and wedge resections [49••]. They report that 38% of their reduction patient cohort had a TLC ratio greater than 1.25 before the transplant and that this patient group faced greater need of post-transplantation ECMO and increased mechanical ventilation and hospital lengths of stay. Survival decreased at the 1-, 3-, and 5-year marks for the resected group. One case study explored the possibility of dividing one donor into two lobar transplants for two small recipients with both surviving at the 3-year follow up [50]. Lobar transplants were also studied by Campo-Canaveral De La Cruz et al., with 75 patients showing no overall survival difference at 1- 3-, and 5-years compared to standard transplants [51].

More work needs to be done to clarify how to proceed in situations in which the graft is mismatched with the recipient. From the ISHLTs 2019 report on the focus theme of size match, there is a remark on the scarcity of literature on this topic despite the importance of size-match when a donor offer is considered for acceptance [52]. Past studies report that either oversized allografts fare better or equivalently in both the short- and long-term [46, 53, 54]. When the ISHLT examined differences in weight, they found that 1-year and 5-year survival in recipients of undersized donors were lower. They also found that undersizing by height was correlated to a lower 1-year survival for recipients who had COPD, alpha-1 antitrypsin deficiency, and cystic fibrosis. This was also true for 5-year survival in the same patient groups but not those recipients with interstitial lung disease (ILD). The report thus concludes that undersizing both height and weight leads to poorer outcomes in all diagnostic groups except for recipients with ILD [52].

Despite the investigative efforts outlined here and the seminal papers that have helped establish the theory behind size mismatch, as lung transplantation is a rapidly evolving field, studies using the latest data centers can provide are needed. Measuring lung volumes based on CT scans has the promise to positively impact the field and could shed greater light on the topic of size mismatch.

## Delayed Chest Closure

However, with grafts that are overly large for the recipient chest, there may be a need to delay chest closure. Delayed chest closure can also be necessary in transplantations with severe bleeding or pulmonary edema [55]. The rate of this is reported to be as high as 29% in some cases but can vary and the question of outcomes is still unclear [56•]. In a retrospective review of 47 propensity matched recipients, there were no differences in pneumonia, deep found infections, or 6-month composite infections between those with delayed chest closure and those without [56•]. There were, however, more transfusions, intubations, and more severe primary graft dysfunction. These patients also remained in the hospital for longer and had worse pulmonary function tests 6 years out, but there were no effects on survival at 6 months, 1 year, and 5 years.

The findings of more severe PGD were supported by a retrospective review by Shigemura et al., who also found higher post-operative bleeding and mortality was associated with the use of delayed chest closure with open skin and retracted ribs [57]. The center had employed three techniques for delayed chest closure with variations on how the ribs and superficial structures were or were not closed. Across the groups, when compared to primary chest closure, there were higher rates of re-exploration and severe PGD. For those who had their skin re-approximated, however, there were fewer incidences of severe PGD requiring post-operative ECMO, decreased mortality, and a shorter ICU stay compared to primary chest closure. In an older study of 28 patients undergoing bilateral transplantation, seven patients with delayed closure were compared to 21 with primary closure with the result of no wound infections in any of these patients [55]. The delayed closure patients were again found to have higher rates of blood transfusion, higher systolic pulmonary artery pressure, higher rate and duration of CPB, and longer lung ischemic time. This compares favorably to the more recent findings by Yeginsu et al. who report that in 16 patients with delayed chest closure, there was a greater time to extubation and a longer ICU stay [58]. Rates of post-operative wound infection were higher but ultimately there was no difference in survival between the two groups.

Similarly, Mohite et al. focused on the technique associated with delayed chest closure and explored approximation of the skin without closure of the sternum and intercostal spaces [59]. This method may avoid the use of ECMO and could be cost-effective. All the aforementioned studies emphasize the role that technique during delayed chest closure may likely play in influencing the outcomes associated with such procedures.

## Ex Vivo Lung Perfusion

Given the severe shortage of donor organs, the application of ex vivo lung perfusion (EVLP) presents as a method for evaluating marginal lungs [60••]. Its development may increase the transplanted donor pool and it holds potential as a timepoint at which therapeutic approaches could be applied to recover an injured lung [61]. The method was originally designed to re-evaluate DCD lungs ex vivo in an extracorporeal membrane oxygenation circuit to allow for the ventilation, oxygenation, and perfusion of an explanted lung [62]. Today, EVLP is used in lung transplant centers worldwide [63–67].

Currently, three major protocols for EVLP are used: the original Lund protocol, the Toronto protocol, and the Organ Care System. Stig Steen and colleagues in the Lund group pioneered the first ex vivo normothermic perfusion system for lungs in the early 2000s [62, 68]. The Lund protocol uses the novel STEEN solution (XVIVO Perfusion AB, Göteborg, Sweden), a buffered extracellular solution with an optimal colloid osmotic pressure for the prevention of pulmonary edema together with erythrocytes. The target flow rate is 100% of cardiac output (CO) in a continuous flow with an open left atrium (LA) [68]. The Toronto group later modified the original protocol, instead opting for an acellular STEEN perfusate and a lower CO against a closed LA. For ventilation, both respiratory rate and FiO2 were reduced to 7/min and 0.21 respectively (compared to 20/min and 0.5). The group have reported using this modified protocol to extend EVLP and preservation time, publishing on normothermic perfusion of up to 12 h [69, 70]. The third protocol, the Organ Care System Lung (OCS) developed by Transmedics (Andover, MA, USA) uses their own OCS Lung Solution together with RBCs and a pulsatile flow with an open LA. Other notable differences include a standardized flow of 2–2.5 l/min and an FiO2 of 0.12 [71].

One of the key differences between the three protocols is the use of cellular vs. acellular perfusate. Several studies have directly compared lung function and performance following the use of either. In a porcine model of extended normothermic EVLP in the organ care system (OCS) which compared acellular to cellular using RBCs and whole blood (WB) protocols, Loor et al. found that only the cellular approaches resulted in clinical standards for transplantation being met at 8 h [72]. After 6 h in acellular perfusion, the lungs showed higher vascular resistance, edema, and worsening compliance. Adding autologous WB to the perfusate showed superior lung preservation compared to the RBCs perfused lungs up to 24 h. In a direct comparison of cellular perfusate and open left atrium to an acellular perfusate and a closed left atrium in swine lungs, Nilsson et al. report lung edema and decreasing compliance after 4 h in EVLP, which were more pronounced in the acellular group [73]. These results point to using cellular perfusates to maximize lung functionality. Despite these preclinical findings, larger human studies have focused on the use of acellular perfusates and have still found favorable outcomes, with Divithotawela et al. reporting no significant difference in time to CLAD between EVLP and non-EVLP recipients [74••].

There have been a few multi-center studies over the years investigating the safety and outcomes of EVLP for standard criteria (SCD) and extended criteria donor lungs (ECD). The NOVEL trial was the first prospective, multi-center, controlled trial and was initially published in 2014 [75] and later updated to include 110 ECD lungs treated with EVLP [76]. The study reported no significant difference in PGD3 rates at 72 h, ICU stay, or 1-year survival compared to standard LTx. In 2018, the INSPIRE trial investigated the use of OCS’s portable EVLP system on SCD lungs and found a decrease in PGD3 rates within the first 72 h as well as no difference in survival at 24 months [77]. This was subsequently followed by the EXPAND trial, utilizing the same protocol on ECD lungs, and similarly found no difference in survival at 24 months post-transplant, however, higher than expected rates of PGD3 were reported [78]. Similarly, the DEVELOP-UK trial, with a modified Lund protocol on ECD lungs, was terminated early due to initial high PGD3 rates and need for ECMO in the EVLP arm within the first 72 h post-transplant [79]. In parallel to the EXPAND trial, PGD3 rates were the highest during the first hours post-transplant but normalized at 72 h and had no detrimental effect on survival, perhaps suggesting a different phenotype of PGD. Other multi-center trials corroborate this, Nilsson et al. reporting on ECD-EVLP cases at two Scandinavian centers using the Lund protocol. No differences were found in long term survival, but PaO2/FiO2 ratios were initially lower and ICU stay was longer in the EVLP cases [66]. In two separate 10-year follow-ups on patients who underwent transplantation with or without EVLP, no significant differences were found between groups in mortality and the incidence of chronic lung allograft dysfunction [74, 80]. Trials investigating the use of initially rejected donor lungs on EVLP report conversation rates as high, or even above 80% [66, 78]. An increase in transplantation volume following the introduction of EVLP has been reported to be 18% in Germany and up to 70% in highly experienced centers such as Toronto, with other centers reporting a significant drop in waitlist times [64, 67, 81].

EVLP has additionally been proposed as a timepoint for therapeutical intervention, and multiple studies have been published on different interventional approaches. Recently, the Toronto group demonstrated a method to convert A antigen donor lungs into universal donor lungs, further increasing the chance of matching a compatible donor and recipient [82]. Mesenchymal stromal (stem) cells and their extracellular vesicles have also been proposed as a therapeutic for reconditioning ECD lungs and ameliorating ischemia-reperfusion injury [83, 84]. Furthermore, novel approaches to improve EVLP are actively being investigated, such as negative pressure ventilation (NPV) and sub-normothermic temperatures to overcome some of the ventilator and metabolic injuries sustained during EVLP [85, 86].

## Conclusion

The brief overview provided here of several prominent topics — bridge to transplant, machine circulatory support, bronchia anastomoses, size mismatch, delayed chest closure, and EVLP — underlines the efforts being made in the field today to further improve upon the results already achieved. While it may appear that there is yet much ground to gain, the substantial strides taken since the dawn of lung transplantation in the early 1960’s prove that such goals are attainable.
